# People with higher interoceptive sensitivity are more altruistic, but improving interoception does not increase altruism

**DOI:** 10.1038/s41598-017-14318-8

**Published:** 2017-11-15

**Authors:** Richard M. Piech, Daniela Strelchuk, Jake Knights, Jonathan V. Hjälmheden, Jonas K. Olofsson, Jane E. Aspell

**Affiliations:** 1 Department of Psychology, Anglia Ruskin University, Cambridge, UK; 2 Department of Psychology, Stockholm University, Stockholm, Sweden

## Abstract

People consistently show preferences and behaviors that benefit others at a cost to themselves, a phenomenon termed altruism. We investigated if perception of one’s body signals – interoception - may be underlying such behaviors. We tested if participants’ sensitivity to their own heartbeat predicted their decision on a choice between self-interest and altruism, and if improving this sensitivity through training would make participants more altruistic. Across these two experiments, interoceptive sensitivity predicted altruism measured through monetary generosity. Improving interoceptive sensitivity did, however, not lead to more altruistic behaviour. We conclude that there is a unique link between interoception and altruistic behaviour, likely established over an individual’s history of altruistic acts, and the body responses they elicit. The findings suggest that humans might literally ‘listen to their heart’ to guide their altruistic behavior.

## Introduction

Despite clear biological and economic advantages of acting in self-interest, people consistently show a range of preferences and behaviors that benefit others at a cost to themselves^1^. We investigated potential physiological mechanisms underlying this phenomenon, known as altruism.

A prominent view of the nature of emotions assigns a critical role to bodily responses^2,3^. The view emphasizes the notion that the bodily response to a situation comes before the feeling - and that the feeling of emotion is the perception of such a response. There is growing evidence that internal bodily states influence emotional experience^4^ and greater sensitivity to internal states is associated with feeling emotions more intensely^5^. Moreover, bodily responses to emotionally-loaded situations bias decisions, and the lack of detectable bodily signals impairs decision making^6,7^. Conversely, improving the monitoring of bodily signals is often emphasized in meditation and emotional intelligence training, practices aimed at improving prosocial interactions^8^.

Interoceptive sensitivity – the accurate detection of internal bodily states – varies markedly between individuals - and continues to be linked to an ever-growing list of affective and cognitive behaviors^9^, including intuitive decision making^10^. The role of interoception in prosocial decision making has recently been examined using the ‘ultimatum game’ in which a proposer decides how to share a given sum of money and the responder chooses whether or not to accept it^11^. Presentation of own heartbeat sounds during the game affected feelings of unfairness and the number of unfair offers by proposers^12^, while delivery of painful (interoceptive) stimuli during the game caused people to make fewer fair offers^13^. By contrast, in meditators, a different network of interoceptive brain areas were recruited than in controls during the game, and the meditators made more rational decisions^14^. Given such links between interoceptive sensitivity, experienced emotions and decision making, it is conceivable that interoceptive sensitivity may mediate the extent to which bodily cues affect prosocial decision making.

## Methods

In the present study, we probed the possible association between the sensing of one’s own body signals and the decision to act altruistically: we investigated if deciding on a choice between self-interest and altruism is related to how well people sense their own physiological body processes (interoceptive sensitivity). To probe altruistic behavior, we used a modified *dictator game* ^15^, in which the player divides real money between herself and another, unknown person, as they please. The game measures pure altruism, and corresponds to real-life charitable giving^16^. As a second index of altruistic behavior, we probed participants’ willingness to help the experimenter with a tedious questionnaire following the completion of the dictator game task and after they had been renumerated for their time. Additionally, we measured how well participants performed on a heartbeat detection task, to obtain a measure of interoceptive sensitivity (experiment 1), and provided training to improve their interoceptive sensitivity (experiment 2). Participants provided self-report measures of materialism, a trait reflecting the importance of personal goals relating to financial success, attractive appearance and social recognition^17^, as such goals may often clash with altruistic behaviors. We also collected self-reported empathy^18^, the ability or tendency to ‘feel what it is like’ to be someone else: to infer another’s emotion, and/or to share, or vicariously feel, the emotion of an other^19,20^ - as some studies have shown it correlates with prosocial behavior^21,22^.

**Figure 1:**
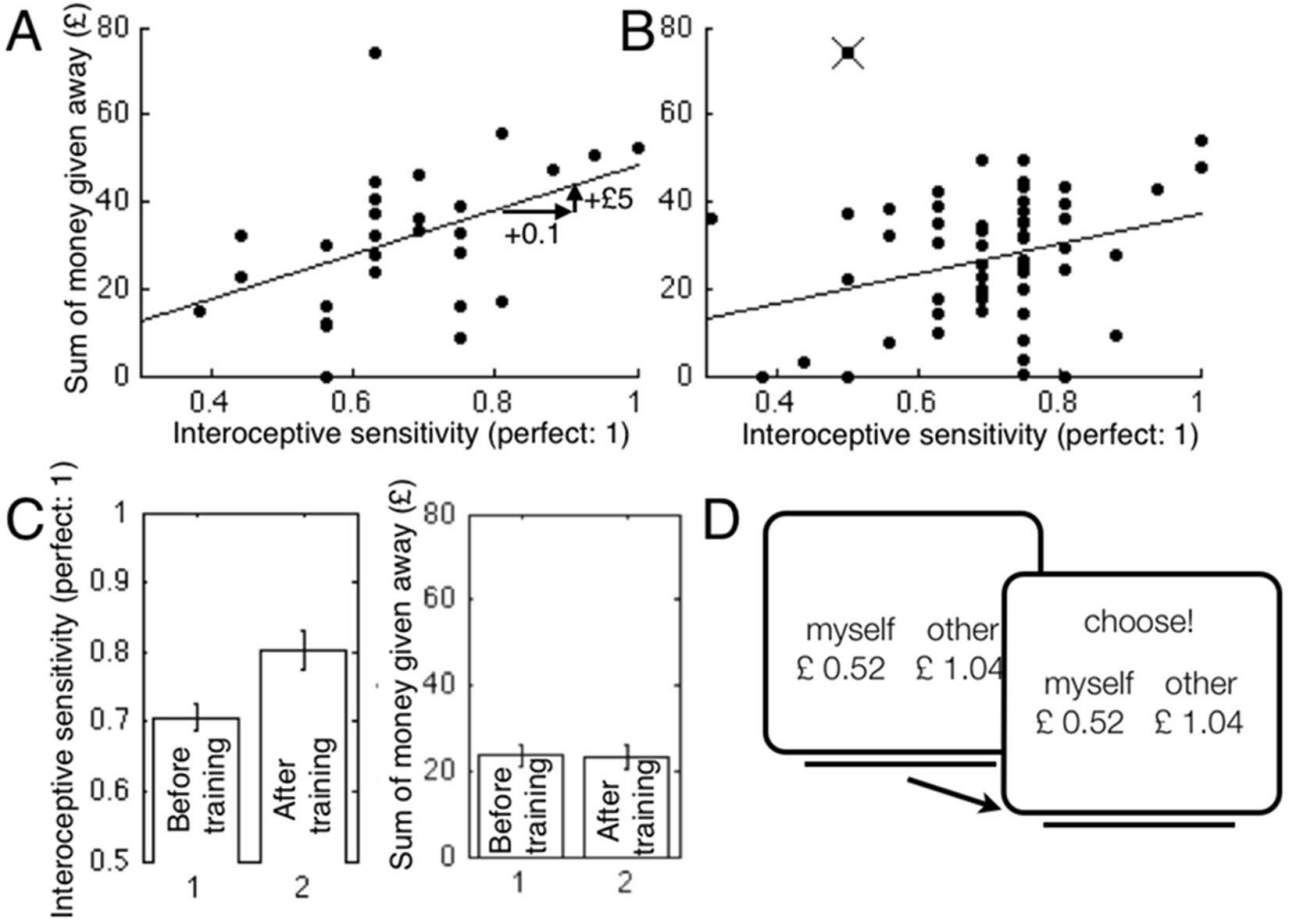
Participants’ monetary generosity increases with interoceptive sensitivity. Sum of money given to another person in dictator game, in pounds (GBP), and interoceptive sensitivity. (**A**) *Interoceptive sensitivity experiment* (experiment 1); (**B**) *interoceptive training experiment* (*experiment 2*). Lines illustrate linear regression functions. Crossed out dot: one participant (perfectly generous, over 3 standard deviations above the mean), excluded from the analysis. (**A**) An improvement in interoception by 10% (a shift of 0.1 to right) corresponds to giving £5 more to another person. (**C**) Improved interoceptive sensitivity does not lead to more giving. Left panel: interoception training improved *interoceptive sensitivity*: mean scores before and after training (t^28^ = 3.22, p = 0.003). Right panel: interoception training does not change *monetary generosity*: mean scores before and after training (t^28^ = 0.21, p = 0.838). (**D**) Illustration of a single trial in the adapted dictator game. Participants choose an amount for themselves or an amount for another person. Amounts and ratios vary (£0.10 to £3.00, ratios 3:1 − 1:3). Two randomly chosen trials are paid out at end (maximal gain £6, minimal gain 0).

In an *interoceptive sensitivity experiment* (experiment 1), 30 participants completed a sequential dictator game after providing informed consent. In the game (Fig. 1D), participants choose one of two amounts of money, to give to oneself or to another, anonymous player. A choice may be: ‘Myself: £0.52 or Other: £1.04’. The amounts vary between £0.10 and £3.00, and the ratios between 3:1 and 1:3. There are 60 such choices. Participants, and the anonymous other player, receive the money allocated on two randomly selected trials at the end of experiment. Altruistic behavior was measured as *monetary generosity*, the sum of money allocated to the other player on all trials. Participants then completed a heartbeat detection task (ref.^23^, see also supplementary materials), in which they listened to a series of beeps synchronous with their heartbeat or to beeps at a control frequency (at an accelerated or decelerated speed). They had to indicate if the beeps they heard were in time with their own heartbeats or not, over 16 trials (half were synchronous and half asynchronous). Interoceptive sensitivity was scored as the proportion of correct answers. Participants then filled in a measure of materialism^17^, and of empathy^18^, before they were paid for participation, and in accordance with their dictator game decisions, and told that they were free to leave. Then, the experimenter said the participant could help him/her by completing a collection of mathematical tasks. Time spent on the tasks was used as a measure of helping behavior.

An *interoceptive training experiment* (experiment 2, N = 57) followed the same procedure, except that one group of participants received *interoceptive training*: after the initial heartbeat detection task, they completed it a second time, this time with feedback (they were told if their answer was correct or not). A third heartbeat detection task was then completed (without feedback) to evaluate the training effect. The control group was trained on a task assessing the detection of audio-visual synchrony as a control procedure (see supplementary materials), and did not perform a second heartbeat detection task.

The studies were approved by the Institutional Research Ethics Review Boards of Anglia Ruskin University and Stockholm University, and carried out in accordance with their guidelines and the Declaration of Helsinki. All participants gave written informed consent prior to participation.

## Results

*Interoceptive sensitivity experiment* (experiment 1, Fig. 1A). As hypothesized, participants’ monetary generosity correlated with *interoceptive sensitivity* (r^28^ = 0.44, p = 0.019, 95% Confidence Interval [0.08, 0.70]), and with *low materialism* (r^28^ = 0.40, p = 0.036, 95% CI [0.03, 0.67]), but not with *empathy level* (r^28^ = 0.18, p = 0.356, 95% CI [−0.21, 0.52]. The time participants spent helping did not correlate with interoceptive sensitivity, low materialism, or empathy, indicating that this measure of altruism is not linked to those factors (see supplementary materials).

Because we aimed to influence interoceptive sensitivity experimentally, we also calculated a linear regression with *only* interoceptive sensitivity as predictor. The regression suggested that a 10% improvement in interoceptive sensitivity would be associated with a £5 increased giving (a 16% increase on the mean of £32.11) to the anonymous other person (Fig. 1A). We aimed to achieve such an improvement in experiment 2.

*Interoceptive training experiment* (experiment 2, Fig. 1B,C). Substantiating the finding from experiment 1, in experiment 2 we also observed that participants’ monetary generosity correlated with *interoceptive sensitivity* (r^56^ = 0.32, p = 0.018, 95% CI [0.06, 0.54]), but, unlike in experiment 1, the significant association was not observed for monetary generosity and *low materialism* (r^56^ = 0.12, p = 0.390, 95% CI [−0.15, 0.37]). As in experiment 1, there was no significant association between monetary generosity and empathy (r^56^ = 0.11, p = 0.427, 95% CI [−0.16, 0.36]).

Participants in the *interoceptive training experiment* (experiment 2) improved their interoceptive sensitivity performance by 10% (t(28) = 3.22, p = 0.003; Fig. 1C), but that improvement did not make them more generous after the training (t(28) = 0.21, p = 0.838; Fig. 1C, see supplementary materials). Unlike in experiment 1, the second measure of altruism, helping time, correlated significantly with interoceptive sensitivity (r_Spearman_(57) = 0.27, p = 0.044), but not with low materialism, or empathy (see supplementary materials).

## Discussion

Across two experiments, interoceptive sensitivity predicted monetary generosity. These results provide evidence for a link between interoception and altruistic behaviour. The link with altruistic behaviour is specific to interoceptive sensitivity, as other measures we obtained did not predict monetary generosity. We can also discount the possibility that the critical property linking altruism and interoception is a *general sensory sensitivity* to body or environmental signals. In a third experiment (*smell sensitivity experiment*), we tested participants’ performance detecting faint smells, a perceptual signal that is intimately linked to the perception of internal and homeostatic states in the generation of food-related behaviors^24^. If altruism were higher in individuals with better overall sensitivity to faint sensory impressions, olfactory detection sensitivity is a plausible candidate to display a pattern of association similar to that observed for interoception. We asked whether smell detection sensitivity would be similarly associated with altruistic behavior in the dictator game. The correlation of smell sensitivity to altruism was much weaker than those obtained in experiments 1 and 2 for interoceptive sensitivity, and statistically not significant [r^21^ = 0.16, p = 0.488, 95% CI [−0.26, 0.54]]. This indicates that altruistic behavior in experiments 1 and 2 is likely to be specifically linked to their interoceptive sensitivity, rather than sensitivity to any faint sensory signal capable of interacting with internal states.

Low materialism shows a trend to predict both forms of altruistic behavior, monetary generosity and helping (see supplementary materials), but not consistently across the experiments. Empathy showed no association with altruistic behaviour, in contrast to some previous studies (e.g.^21,22^), and this may be due to our particular empathy measure: the empathy quotient questionnaire^18^ which measures both affective and cognitive empathy. Had we measured cognitive and affective empathy separately, e.g. with the Questionnaire of Cognitive and Affective Empathy^19^ and the Multifaceted Empathy Test^20^, a relation might have been found.

The *interoceptive sensitivity experiment* points to an *association* between interoceptive sensitivity and altruistic behavior but cannot demonstrate causality. The *interoception training experiment* tested one possible form of causation. Its result shows that enhancing the ability to detect one’s heartbeat, through short training, does not enhance altruistic behavior in a subsequent game.

If there indeed was a causal relationship between sensitivity to heartbeats and altruistic behavior (which our *interoception training experiment* fails to demonstrate), what could be its nature? The relationship might be based on short-term signals, reflecting moment-to-moment changes in the body. Such a relationship would be consistent with a value-based model of decision making^25^, whereby peripheral signals provide value-weights to guide decisions, or with the *somatic marker hypothesis* of behavior guidance^6^. A person might encounter an emotionally evocative situation (like a choice to give away money, or not), that causes a heartbeat signal change (e.g. a strengthening of the signal). That bodily signal is interoceptively detected, and biases the choice towards the altruistic option (the *direction* remains unexplained, but could relate to avoiding the social stress of not sharing^26^). Peripheral physiological events have been shown to influence complex cognitive processes on a short time scale (e.g. heartbeat to heartbeat^27^; giving such a scenario some plausibility. But the results of our *interoceptive training experiment* show that enhancing the body signal detection through training does not bias choice - it does not make people more altruistic in a subsequent game. This result appears inconsistent with a moment-to-moment account that assumes a direct causal link from interoception to altruism (see supplementary materials). What would explain such a result? One possibility is that our procedure is not sufficiently sensitive to the effect.

A possible alternative account focuses on the perception of distress in others. (It supposes that in the course of the dictator game, participants form a representation of distress in the recipient with whom one does not share.) Altruistic people stand out in their ability to detect distress in faces^28^. Moreover, extreme altruists (non-directed organ donors) have larger amygdalae (nuclei activated by emotional material), which also respond more strongly to faces communicating distress than amygdalae of control participants^29^. Given that different levels of interoceptive sensitivity are linked to differences in emotional experience in general^10^, and that body responses specifically contribute to the perception of distress^30^, heightened interoceptive sensitivity may enhance perception of distress and support altruism, explaining the results of our experiments. At this stage, the idea is a speculation which may be confirmed in studies including measurement of stress and distress, which our studies do not. Additionally, the functional significance of the amygdala for fear and anxiety may connect that structure to the complex of interoception and altruism. This remains to be explored in future studies. Future studies may also focus on the role emotions and their components (e.g. activation) experienced during the task play in altruistic behaviour.

It is worth noting that although we have assumed that our heartbeat discrimination task reflects interoceptive sensitivity specifically, it is in fact measuring the brain’s ability to integrate or compare exteroceptive (auditory) and interoceptive (cardiac) stimuli to make synchronicity judgments, and thus performance on this task is likely to be dependent on a somewhat different network of brain areas than the ‘cleaner’ heartbeat tracking task^31^, which requires participants to internally count heartbeats. We chose the heartbeat discrimination task, however because it is not affected to the same degree by confounds such as time estimation ability and beliefs about one’s own heart rate^32^. Both tasks are problematic, also because they often fail to correlate with each other, and with interoceptive awareness^33^, particularly in relatively small samples of poorly performing participants. Although differences in performance on our heartbeat discrimination task could be due to variations in attention^34^, our failure to find a significant association between prosocial behavior and smell sensitivity (see supplementary material), which should also be affected by attention, contradicts this argument.

Our results suggest that altruistic acts may be influenced by the representation of one’s body in the brain. Although the mechanisms underpinning this relationship need to be clarified, the present findings indicate that humans in some sense ‘listen to their heart’ to shape their altruistic behaviors.

## Electronic supplementary material

Supplementary Information
