# Supplementary material for: People with higher interoceptive sensitivity are more altruistic, but improving interoception does not increase altruism
Source: Sci Rep. 2017 Nov 15;7:15652. doi: 10.1038/s41598-017-14318-8 (PMC5688159; doi:10.1038/s41598-017-14318-8)
Supplement: Supplementary file 1 — Supplementary Information [file 41598_2017_14318_MOESM1_ESM.pdf]

People with higher interoceptive sensitivity are more altruistic, but improving interoception does not increase altruism.

Authors: Richard M. Piech[1,2]*, Daniela Strelchuk[1], Jake Knights[1], Jonathan V. Hjälmheden[2], Jonas K. Olofsson[2], Jane E Aspell[1].

Affiliations:

[1]Department of Psychology, Anglia Ruskin University, Cambridge, UK.

[2]Department of Psychology, Stockholm University, Stockholm, Sweden.

*Correspondence to: RMP: richard.piech@anglia.ac.uk

Supplementary Materials:

Materials and Methods

Results

Figures S1, S2

Discussion and Limitations

References

Materials and Methods:

The experiments were approved by the institutional ethics review boards. Participants experiment 1: recruited from student and community population in Cambridge (UK); N=30; 18 female; heartbeat task failed for 2; help task time was not recorded for 6; Aspiration Index was not recorded for 2; additionally 6 pilot participants. Participants experiment 2: recruited as above; N=57 (29 (21 female) training condition, 28 (15 female) control condition); dictator game failed for 2. One participant allocated all the money to the other player in dictator game 1 (before training). The allocation was thus over 3 standard deviations above the mean, and the participant was excluded from the analysis (crossed out dot in Figure 1). Participants experiment 3: recruited from student and community population in Stockholm (Sweden). N=21, 16 female.

Participants were compensated for their time, and in line with their (and another player's) decisions in the dictator game. Experiment 1 sequence: participants were informed about, and consented to study participation; they performed the dictator game, the heartbeat detection task, answered survey questions, were paid and told they are free to leave, were then offered an opportunity to help the experimenter. Experiment 2 sequence: as in experiment 1, with a different heartbeat detection procedure: a first heartbeat detection task; then heartbeat detection training, then a second heartbeat detection task; then a second dictator game task. Experiment 2 sequence for the control group: first they performed the dictator game, then they performed an audio-visual synchrony discrimination (A-V) task, then they performed the A-V task again, with feedback, then they performed the A-V task again, without feedback, then they performed the dictator game for a second time and finally they

performed the heartbeat detection task. Experiment 3 sequence: dictator game; other tasks (not reported here); smell sensitivity test; questionnaires (not reported here).

Dictator game task

The dictator game is based on a task described by Zaki and Mitchell *(1)*. During this computer delivered task, participants choose between two options, for example: 'myself: £0.52' - 'other: £1.04', over a number of trials. Choosing 'other' means another person (real, but anonymous) will get £1.04 (if the trial is later randomly selected as a paid trial; there are two paid trials out of 60). The sums vary between £0.10 and £3.00, and their ratios are: 3:1, 2:1, 3:2, 4:3, 5:4, 1:1, 4:5, 3:4, 2:3, 1:2, 1:3. Thus, the larger amount is on some trials assigned to the decider ('favourable'), on others to the other person ('unfavourable', *15*; our main text fig 1D). There are equal numbers of these two types of trials (favourable and unfavourable). The sum of money allocated to 'other' corresponds to monetary generosity and is an index of altruistic behavior. Unbeknownst to the participant, the 'other' person is the next participant in the study. As payment for the game, each participant receives the amount from two randomly selected trials from playing their own game, according to their own choice. They also receive the amount from 2 randomly selected trials from the previous participant's game, according to that participant's choice. The task consisted of 12 practice trials and 60 experimental trials.

Heartbeat detection task and training

During the heartbeat detection (discrimination) task *(2)*, on each trial, participants listened, via laptop speakers, to a series of 20 auditory beeps simultaneous with their own heartbeat, recorded with electrocardiogram (ECG) electrodes on the chest. ECG

provided the input for in-house developed ExpyVR software (http://lnco.epfl.ch/expyvr), which produced the brief auditory beeps, triggered by the R-wave of the ECG in the synchronous condition, or it produced beeps at a speed of either 80% or 120% of the frequency of the participant's preceding two R-waves to create the asynchronous condition. Participants had to indicate on each trial if the beeps were in time with their own heartbeat or not. There were 16 trials. The proportion of correct answers indicates how good a person is at sensing their own heartbeat, and is an index of interoceptive sensitivity.

Training and control condition

In the *interoceptive training experiment* (experiment 2, N=57), one group of participants received interoceptive training: after the initial heartbeat detection task, they completed it a second time, this time with feedback (they were told, after their response to each trial, if their answer was correct or not). A third heartbeat detection task was then completed (without feedback) to evaluate the training effect. The control group were trained on a task assessing the detection of audio-visual synchrony as a control procedure. They saw geometric shapes appear on the computer screen. At the same time, they listened to sounds (beeps, as in the experimental condition) via the laptop speakers. Their task was to report if the visual shapes on the screen appeared at the same time as the auditory signals. They were given correct feedback on the answers in the training block, and all parameters were kept the same as for the experimental condition. The control group did not perform a second heartbeat detection task.

Questionnaires

Participants responded to questions from the Aspiration Index *(3)*, a measure of materialism *(4)*. It classifies a person's goals and values into 'intrinsic' and 'extrinsic' ones and indicates how much importance is given to each. (Intrinsic goals: Self-Acceptance, Affiliation, Community Feeling, Physical Fitness; extrinsic goals: Financial Success, Attractive Appearance, Social Recognition.) A relatively high importance assigned to intrinsic goals corresponds to low materialism. Participants also responded to questions from the Empathy Quotient for adults *(5)*.

Help task

After finishing all other components of the study, receiving payment, and being told that they are 'free to go', participants were asked by the experimenter for help with a different study. The help takes the form of filling in as much as possible of a questionnaire, which consists of 25 pre-university level mathematics test questions. Time spent filling in the questionnaire provides a measure of helping behaviour and an index of altruism. The proportion of items of the questionnaire attempted is a second measure of helping behaviour. We report the analysis based only on time spent helping, as it is less dependent on personal mathematical skills and interest in mathematics. Analyzing the proportion of items completed does not lead to different conclusions.

Smell sensitivity test (Experiment 3)

We measured the threshold at which participants were able to detect weak odors *(6)*. The odor was n-butanol, applied at 16 concentrations (1/16 concentration to 1/1 concentration). Smell sensitivity was operationalized as the lowest concentration that could be reliably detected. Participants where required to wear a blindfold; they were

given sets of three pens (one at a time, pens were designed to dispense odors) to smell (held by the experimenter at a constant distance under the nose). Participants were asked to identify which of the three pens contained the odor (the other two were odorless). The test began with the weakest concentration. Every time the participant failed to identify the correct sniffing stick, they moved to the next odor concentration set. If they identified the odor correctly, the test was repeated at the same concentration level. The odor was considered reliably detected when a participant identified it correctly four times in a row. Participants were given a strong (1/1) and medium (1/8) strength test smell of n-butanol as reference before the experiment. Possible scores were: 16 (perfect; lowest concentration detected reliably) – 0 (failed to detect strongest concentration).

Results

Dictator game

Experiment 1. On average, participants allocated to themselves (summarized over all trials of the task) GBP 44.39, ranging from 71.96 to 1.51, and to the other player GBP 32.11, ranging from 74.03 to 0.

Experiment 2. Game 1: participants allocated to themselves GBP 49.19 (74.04-0), and to the other player GBP 28.85 (73.91-0); Game 2: participants allocated to themselves GBP 52.90 (74.04-3.97), and to the other player GBP 26.24 (70.01-0).

Experiment 3: On average, participants allocated to themselves SEK 337 (~£30), and to the other player SEK 216.

Help task

Experiment 1. Participants completed an average of 24% of the questions (6/25 questions; range: 0-22), taking on average 6.6 minutes (range 0-18.3 minutes; data available for 24 participants). Experiment 2. Participants completed an average of 48% of the questions (12/25 questions; range: 0-25; training group 48%, no-training group 48%), taking on average 16 minutes (range 0-70 minutes; training group 15 minutes, no-training group 18 minutes).

Heartbeat detection task and training

In the 16-trial task, identifying one's heartbeat correctly on all trials corresponds to the perfect score of 1. A score of around 0.5 indicates chance performance.

Experiment 1: Mean score was 0.67 (range: 1.0-0.38).

Experiment 2: Mean score in the training group before training was 0.70 (0.88-0.44); and after training 0.80 (1.0-0.50). Mean score for the no-training group was 0.69 (1.0-0.31).

Questionnaires

Aspiration Index (AI). The scores indicate the average importance given to intrinsic or extrinsic values, compared to the average overall importance of goals. A higher number reflects higher importance. High importance of intrinsic values corresponds to low materialism.

Experiment 1. Participants gave intrinsic values an importance of 0.65 (ranging 0.02-1.42), and extrinsic values of −0.86 (ranging −1.89 - −0.03). Data available for 28 participants.

Experiment 2. Participants gave intrinsic values an importance of 0.67 (ranging 0.02-1.42), and extrinsic values of −0.89 (ranging −1.89 - −0.03).

Empathy Quotient. Experiment 1. Participants had an average EQ score of 41.4 (range 19-63). Experiment 2. Participants had an average EQ score of 44.0 (range 18-65).

Smell sensitivity test (Experiment 3, see note *17*)

Mean smell sensitivity was 7.04 (*SD*=3.82). Possible scores: 16 (perfect; lowest concentration detected reliably) – 0 (failed to detect strongest concentration). The correlation of smell sensitivity with money given away was weaker than for interoceptive sensitivity in experiments 1 and 2, and statistically not significant [r(21) = .16, p = .488, 95% CI [-.26, .54]].

Regression: Experiment 1

In addition to the main analysis reported in the manuscript, we conducted a regression which includes all three factors simultaneously. Specifically, we conducted a multiple regression to test if *monetary generosity* is predicted by: heartbeat sensitivity, low materialism (importance of intrinsic values), empathy level. Using the enter method we found that the model explains a significant amount of variance in generosity [F[3,24] = 3.58, p = .029, R2 = .31. R2adjusted = .22]. Heartbeat sensitivity significantly predicts monetary generosity [ß = .38 t[24] = 2.17, p = .040], low materialism predicts monetary generosity missing the significance cut-off [ß = .34

t[24] = 1.95, p = .063], empathy level does not predict monetary generosity [ß = .03 t[24] = .17, p = .86].

Pearson correlations of monetary generosity (with two-tailed p-values and 95% confidence intervals, normality assumption met):
with interoceptive sensitivity [r(28) = .44, p = .019, 95% CI [.08, .70]];
with low materialism [r(28) = .40, p = .036, 95% CI [.03, 0.67]];
with empathy level [r(28) = .18, p = .356, 95% CI [-.21, 0.52]].

CIs were computed based on r-z' transformation, using: CI_95 = z'(r) -/+ (1.96*(1/sqrt(N-3)), and then transformed back to r.

A linear regression with only interoceptive sensitivity as predictor (not accounting for other factors) resulted in the function: sum_to_other = 50.75 x interoception - 2.44. This suggests that a sensitivity improvement of 1.0 would result in giving £50.75 more, or an improvement of 10% in giving £5 more (a 16% increase on the mean of £32.11) to the anonymous other person (fig. 1 A).

We conducted a further multiple regression to test if *helping time* is predicted by: heartbeat sensitivity, low materialism, empathy level. Using the enter method we found that the model does not explain a significant amount of variance in helping time [F[2,17] = 1.63, p = .214].

Spearman's rho (rS) correlations of helping time were (with two-tailed p-values; normality assumption was not met for the 'helping time' variable, with many participants helping a little, and longer times relatively rare):

with heartbeat sensitivity [rS(24) = .14, p = .530];

with low materialism [rS(24) = .34, p = .106];

with empathy level [rS(24) = .21, p = .331];

Regression: Experiment 2

We repeated a multiple regression as in experiment 1, including participants from both groups, to predict monetary generosity. We used the monetary generosity score from the first dictator game (before treatment) and heartbeat sensitivity from the first measurement (before treatment), to confirm findings from experiment 1. The multiple regression tested if monetary generosity is predicted by: interoceptive sensitivity, low materialism, empathy level. Using the enter method we found that the model misses significance when attempting to explain the amount of variance in monetary generosity [F[3,50] = 2.26, p = .093]. We therefore removed the predictor shown in the analysis of experiment 1 and this analysis as the weakest from the model (empathy; [ß = .03 t[49] = .18, p = .86]. The resulting multiple regression tested if monetary generosity is predicted by: interoceptive sensitivity, low materialism. Using the enter method we found that the model explains a significant amount of variance in monetary generosity [F[2,51] = 3.44, p = .040, R2 = .12. R2adjusted = .08]. Interoceptive sensitivity significantly predicts monetary generosity [ß = .32 t[51] = 2.46, p = .017], importance of intrinsic values does not predict monetary generosity [ß = .12 t[51] = .94, p = .350].

Pearson correlations of monetary generosity (with two-tailed p-values and 95% confidence intervals, normality assumption met):

with interoceptive sensitivity [r(56) = .32, p = .018, 95% CI [.06, 0.54]];

with low materialism [r(56) = .12, p = .390, 95% CI [-.15, 0.37]];

with empathy level [r(56) = .11, p = .427, 95% CI [-.16, 0.36]];

Monetary generosity and split-half interoception groups.

As a complementary analysis to the correlations shown in the main text (Fig. 1A, 1B), we split all participants from experiments 1 and 2 into two groups, based on their interoceptive sensitivity score, and compared the sum donated for the groups. Participants classified as low interoception group donated less than participants from the high interoception group (£27.02 (SE: 2.14) versus £31.39 (SE: 2.21), see Fig. S1).

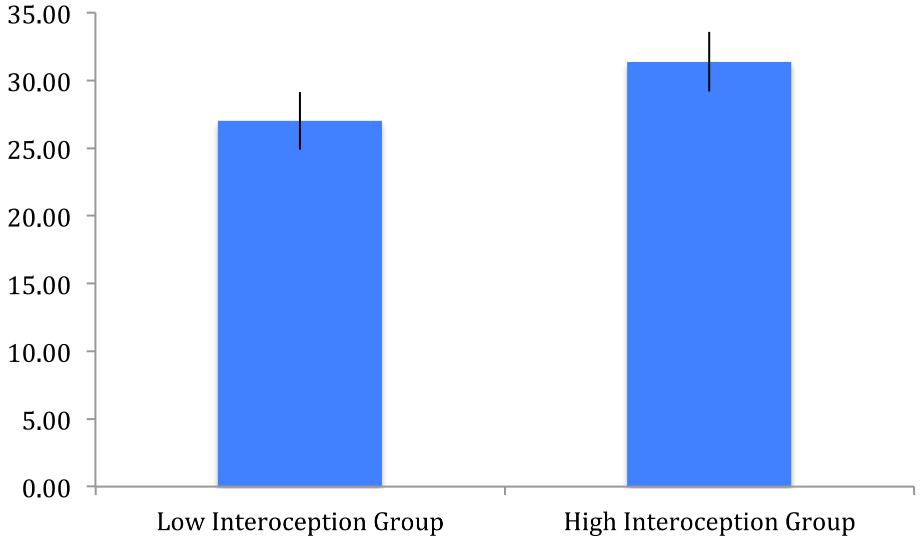

Figure S1: Mean amount donated (y-axis, in GBP) by participants classified as low and high interoception groups based on a split-half analysis of their interoceptive sensitivity score. Participants from Experiment 1 and 2 are included. Error bars indicate standard error of the mean.

Help task

We repeated a multiple regression as in experiment 1, including participants from both groups, to predict helping time. We used the interoceptive sensitivity from the first measurement (before treatment), to confirm findings from experiment 1. The multiple regression tested if helping time is predicted by: interoceptive sensitivity, importance of intrinsic values, empathy level. Using the enter method we found that the model does not explain a significant amount of variance in helping time [$F[3,53] = 1.59$, $p = .203$].

Spearman's rho (rS) correlations of helping time were (with two-tailed p-values; normality assumption was not met for the 'helping time' variable, with many participants helping a little, and longer times relatively rare):

with interoceptive sensitivity [$rS(57) = .27$, $p = .044$];
with low materialism [$rS(57) = .20$, $p = .130$];
with empathy level [$rS(57) = -.07$, $p = .618$].

Heartbeat detection task training

We conducted a paired-samples t-test to check if the training had been successful. It revealed that heartbeat sensitivity performance significantly improved after the

training, with the mean being 10% higher (Mbefore = .7055; SDbefore = .10; Mafter = .8045; SDafter = .15 ; $t(28) = 3.22$, $p = .003$).

To test if this increase corresponded to more generosity, we conducted a paired-samples t-test on dictator game amounts allocated to other player before and after the heartbeat detection training (only for participants who underwent the training). The test revealed that there was no significant difference, with the small mean difference pointing in the opposite (to hypothesis) direction (Mbefore = 23.6; SDbefore = 13.7; Mafter = 23.3; SDafter = 15.3 ; $t(28) = .21$, $p = .838$).

It is conceivable that while generosity in the training group remained flat, it could have dropped significantly in the no-training group. To test this, we conducted an analysis of variance testing the dictator game scores before and after training for both participant groups. The results indicate that the groups do not differ in their monetary generosity overall, nor that changes between pre- and after training are different for the two groups [no difference between groups: $F(1,54) = 2.46$, $p = .123$; no interaction: $F(1,54) = 1.25$, $p = .268$]. Nominally, the no-training group did indeed drop more than the training group: scores training group: Mbefore = 23.6, SDbefore = 13.7, Mafter = 23.3, SDafter = 15.3; Scores no-training group: Mbefore = 30.7, SDbefore = 14.1, Mafter = 27.8, SDafter = 14.6. (See Figure S2).

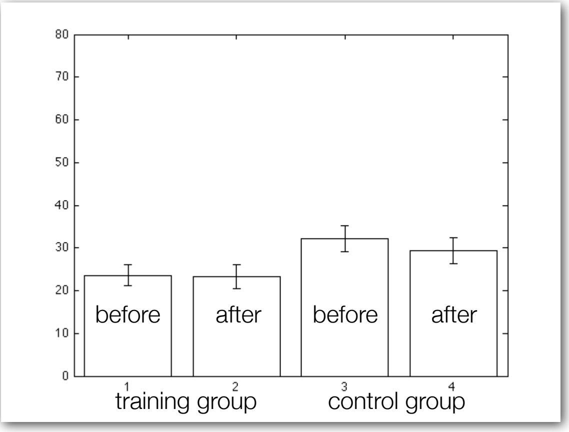

Figure S2: Experiment 2: effect of interoception training on *monetary generosity*: mean scores before and after training, for the experimental group (training group) and control group; y-axis: sum of money given to another person in dictator game, in GB pounds (£); bars: SEM; [data for the experimental group are identical to Figure 1C, right panel, in main text].

Sex

We had no hypotheses about how the relationship between monetary generosity and the other factors recorded might differ in the sexes, and the reported analysis reflects this. For exploration, we conducted additional analyses with sex of the participant as a predictor. This did not contribute significantly to the explained variance of the models, or change the results in a meaningful way:

Regression for monetary generosity in experiment 1 (including sex): [F[4,23] = 2.70, p = .056, R2 = .32. R2adjusted = .20]. Thus, adding sex as a predictor means the overall model narrowly misses the significance level. The contribution of the three factors of interest remains the same (interoception: clear contribution; low materialism: marginal contribution; empathy: no contribution).

The regression model for helping time in experiment 1 remains not significant after including sex: [$F[4,19] = 1.21$, $p = .338$, $R2 = .20$. R2adjusted = .04].

Regression for monetary generosity in experiment 2 (including sex):
[$F[3,50] = 2.56$, $p = .065$, $R2 = .13$. R2adjusted = .08]. Thus, adding sex as a predictor means the overall model narrowly misses significance. The contribution of the two factors of interest remains the same (interoception: clear contribution; low materialism: no contribution).

The regression model for helping time in experiment 2 remains not significant after including sex: [$F[3,53] = 1.49$, $p = .227$, $R2 = .08$. R2adjusted = .03].

Baseline heart rate

Performance on the heartbeat detection task is better for participants with lower baseline heart rate [Experiment 2 Pearson correlation: [$r(57) = -.29$, $p = .027$, 95% CI [-.52, -.04]];]. This does not explain our results: in experiment 2, the regression model for generosity misses significance when baseline heart rate is added as a predictor [$F[3,50] = 2.29$, $p = .090$, $R2 = .12$. R2adjusted = .07]. The contribution of the two factors of interest remains the same (interoception: clear contribution; low materialism: no contribution).

Smell sensitivity experiment

To test whether the association between altruism and interoception generalized to the detection of other faint sensory signals, we investigated olfactory detection thresholds. Olfaction is intimately linked to internal states, as it is well-established

that olfactory sensitivity and perception are modulated by hunger/satiety states (*7*). We tested 21 participants' performance detecting smells according to a well-established protocol (*6*), as well as their behaviour in the dictator game. The correlation for smell sensitivity was much weaker than for interoceptive sensitivity in experiments 1 and 2, and statistically not significant [r(21) = .16, p = .488, 95% CI [-.26, .54]]. This shows that link between altruistic behaviour and interoception shown in experiments 1 and 2 is unlikely to generalize to other perception modalities, and we therefore propose the link to altruistic behaviour might be specific to interoception.

Olfactory sensitivity and interoceptive sensitivity

For technical reasons, the HB detection task used together with smell sensitivity was different from the main experiments (1 and 2), the 'counting task' by Schandry *(8)*. The correlation between that task and smell sensitivity is negative (non-significant), r(20)=-.12, p=.62.

Discussion and Limitations

In our experiments, we used heartbeat detection performance as an index of interoception. While heartbeat detection is an interoceptive process, and different interoceptive processes share neuroanatomical features *(9)*, we don't currently have a sufficient amount of evidence to confirm that in humans, performance in this task is indicative of interoceptive ability in general, which encompasses other interoceptive processes (*10*). Future studies should address the generalizability of interoceptive performance, and its potential role in altruism.

It is conceivable that compliance of participants contributed to our initial results, in addition to the effect of interoceptive sensitivity. Non-compliant participants may expend less effort on the heartbeat detection task, and subsequently expend less effort on being altruistic. While this is possible, one would expect such a compliance effect foremost in the help task, and to a lesser degree in the dictator game. Data do not support this, as the association was stronger for the dictator game. Further, only 4 out of 29 participants in the training group in experiment 2 did not improve or did not retain a high interoceptive sensitivity level - suggesting that low compliance was not a problem.

In contrast to our findings for monetary generosity, performance in the 'help' task was not predicted by interoceptive sensitivity, empathy or materialism levels. One reason for this may be that the help task is a cruder measure of altruism and subject to more confounds than the dictator game performance: time spent helping was likely influenced by how much participants liked doing maths, maths ability, whether they had a subsequent appointment, their opinion of the experimenter, etc. The dictator game and the help task also differ in many important ways - in the former, the beneficiary is unseen and unknown and money rather than time is at stake – and may have different brain bases.

That less materialistic people may also act more generously is a promising thesis in need of further investigation. Because materialism has malleable components even in highly materialistic individuals *(11)*, this presents an opportunity for interventions. Such interventions could stimulate people to address 'bigger than self' problems, e.g.

regarding the climate or poverty, which are difficult to solve with appeals to self-serving motives *(12).*

References

1. J. Zaki, J. P. Mitchell, Equitable decision making is associated with neural markers of intrinsic value. Proc. Natl. Acad. Sci. U.S.A., **108(49)**, 19761-19766 (2011).

2. W. E. Whitehead, V. M. Drescher, P. Heiman, B. Blackwell, Relation of heart-rate control to heartbeat perception. *Biofeedback Self. Regul.*, **2(4)**, 371–392 (1977).

3. T. Kasser, R. M. Ryan, Further examining the American dream: differential correlates of intrinsic and extrinsic goals. *Personal. Soc. Psychol. Bull.* **22** 280–87 (1996).

4. T. Kasser, Materialistic Values and Goals. *Annu. Rev. Psychol.* **67**, 489-514 (2016).

5. S. Baron-Cohen, S. Wheelwright, The empathy quotient: an investigation of adults with Asperger syndrome or high functioning autism, and normal sex differences. *J. Autism Dev. Disord.*, **34(2)**, 163-175 (2004).

6. T. Hummel, G. Kobal, H. Gudziol, A. Mackay-Sim, Normative data for the "sniffin' sticks" including tests of odor identification, odor discrimination, and olfactory thresholds: an upgrade based on a group of more than 3,000 subjects. *Eur Arch Otorhinolaryngol* **264**:237–243 (2007).

7. M. R. Yeomans, Olfactory influences on appetite and satiety in humans. *Physiology & Behavior*, ***87***(4), 800-804 (2006).

8. R. Schandry, Heart beat perception and emotional experience. *Psychophysiology,* 18, 483-488, (1981).

9. A. D. Craig, How do you feel? Interoception: the sense of the physiological condition of the body. *Nature Rev Neurosci*, ***3*****(8)**, 655-666 (2002).

10. S. N. Garfinkel, A. K. Seth, A. B. Barrett, K. Suzuki, H. D. Critchley, Knowing your own heart: distinguishing interoceptive accuracy from interoceptive awareness. *Biol Psychol* **104**, 65-74 (2015).

11. P. Chilton, T. Crompton, T. Kasser, G. Maio, A. Nolan (2012). Communicating bigger-than-self problems to extrinsically-oriented audiences. *Common Cause Research, UK*.

12. M. J. Sandel, *What money can't buy: the moral limits of markets*. Macmillan (2012).
